# Validity and reliability of a performance evaluation tool based on the modified Barthel Index for stroke patients

**DOI:** 10.1186/s12874-017-0409-2

**Published:** 2017-08-25

**Authors:** Tomoko Ohura, Kimitaka Hase, Yoshie Nakajima, Takeo Nakayama

**Affiliations:** 1 Division of Occupational Therapy, Faculty of Care and Rehabilitation, Seijoh University, 2-172 Fukinodai, Tokai, Aichi, 476-8588 Japan; 2 Department of Physical Medicine and Rehabilitation, Kansai Medical University Hospital, 2-3-1 Shinmachi, Hirakata, Osaka, 573-1191 Japan; 3 Clinical Development Center, Asahi Kasei Pharma Corporation, 1-105 Kanda Jinbocho, Chiyoda-ku, Tokyo, 101-8101 Japan; 4 Department of Health Informatics, Kyoto University School of Public Health, Yoshida-Konoe-cho, Sakyo-ku, Kyoto, 606-8501 Japan

**Keywords:** Activities of daily living, Validity, Reliability, Stroke

## Abstract

**Background:**

The Barthel Index (BI) is a measure of independence in activities of daily living (ADL). In the modified Barthel Index (MBI), a five-point system replaced the original two or three or four point rating system. Based on this modified measure, the performance evaluation tool MBI (PET-MBI) was developed in Japan. Although the reliability and validity of PET-MBI have been verified for older people, the use of this tool in stroke patients has not been evaluated. This study investigated the validity and reliability of PET-MBI for stroke patients.

**Methods:**

Ten raters independently determined the BI and PET-MBI scores of stroke patients by direct observation. These patients’ ADL were videotaped, and 10 other raters then evaluated the videos privately and assigned PET-MBI scores twice, one month apart. The criterion-related validity of the PET-MBI against the BI was evaluated using the correlation coefficients for their total scores. Furthermore, to assess inter- and intra-rater reliabilities from the results of the first and second sessions, Fleiss’ intraclass correlation coefficients (ICCs) were calculated for the total scores, with the lower limits of the 95% confidence interval (95%CI), along with weighted kappa (κ_w_) coefficients for agreement in individual tasks of this evaluation tool. ICC and κ_w_ coefficients of 0.81–1.00 were considered to be “almost perfect” agreement.

**Results:**

The mean age of the 30 patients (23 men, 7 women) was 71.9 (standard deviation 10.5) years. One patient had diplegia, 14 had right hemiplegia, and 15 had left hemiplegia. For the total scores obtained by direct evaluation, Pearson’s and Spearman’s correlation coefficients of the BI versus the PET-MBI were both 0.95 (lower limit of the 95%CI, 0.90). The ICC representing inter-rater reliability for the first session was 0.99 (lower limit of the 95%CI, 0.98]. For intra-rater reliability, the mean value of the ICCs was 0.99 (range, 0.99–1.00). For individual tasks of the PET-MBI, inter-rater κ_w_ coefficients for the first session ranged from 0.77 to 0.94, with intra-rater κ_w_ coefficients from 0.85 to 0.96.

**Conclusions:**

PET-MBI showed strong criterion-related validity against the BI, with high reliabilities. This scoring system may become a convenient tool allowing anyone to assess ADL.

## Background

A stroke is a life-threatening medical emergency, but if patients survive a stroke, they may still face prolonged difficulties in activities of daily living (ADL) due to severe brain damage. Since many different symptoms and conditions can develop after a stroke, multidisciplinary rehabilitation is critical for people recovering from this disease [1]. In applying rehabilitation therapies to patients with stroke (as with any other disorder), goal setting is essential, and to measure progress in achieving the goals, outcome measurement tools are necessary [2]. Accurate assessment of stroke patients’ ADL greatly helps evaluate the efficacy of stroke medications and rehabilitation. In fact, in measuring the progress of stroke rehabilitation, the Japanese Guidelines for the Management of Stroke [3] recommend implementing ADL assessment scales (in addition to overall, motor function, and muscle tone scales) that have been demonstrated to be reliable and valid. A variety of ADL assessment scales has been developed for stroke patients, as well as for patients with other diseases and conditions. These include the Barthel Index (BI) [4], the modified BI (MBI) [5–8], the Functional Independence Measure (FIM) [9, 10], the Stroke Impairment Assessment Set (SIAS) [11], and the modified Rankin Scale (mRS) [12]. Each of these tools has different advantages and disadvantages. For example, some of them are easy to use, but not as detailed as the others. Some are sensitive to changes, but require advanced knowledge for their administration.

The BI [4] was originally established for assessing the ADL of stroke patients and has been widely used for this purpose. Several groups have developed revised versions [5–8]. In particular, the MBI, created by Shah et al. [5], was developed to achieve greater sensitivity, and its internal consistency has been confirmed for use among stroke patients. The Japanese version of the performance evaluation tool based on the MBI (PET-MBI) was created with permission from the authors of the original MBI (including a back-translation process against Shah et al.’s MBI), and its reliability and validity were then verified in a study targeting 110 elderly individuals requiring care residing in care facilities [13]. In addition, the factorial validity of the PET-MBI for 126 elderly individuals requiring care living at home has also been verified [14].

However, it is still unclear if the PET-MBI can be used as a functional assessment instrument for patients with stroke. So far, the reliability of this tool has only been verified in studies limited to elderly individuals requiring care in which inter- and intra-rater reliabilities were assessed by only two raters. Thus, the reliability of the PET-MBI has not yet been rigorously tested. Furthermore, the concurrent validity of this tool against the established ADL assessment scales is unknown. To address these issues, the reliability and concurrent validity of the PET-MBI for stroke patients were examined in this study.

## Methods

The aim of the research was twofold: 1) validity study, in which the concurrent validity of the PET-MBI (ADL performance) against the BI (ADL capacity) was examined by obtaining the ADL scores of these two measures in stroke patients using direct observation techniques (although this was within the same one-week period, raters evaluated BI in the rehabilitation room, and MBI in daily situations outside the rehabilitation rooms. Therefore, the observations did not pertain to the same scenes); and 2) reliability study, in which stroke patients’ ADL was videotaped, and following editing of these videos, they were evaluated by raters using the BI (ADL capacity) and the PET-MBI (ADL performance). The video raters belonged to different medical institutions from those that conducted the direct examination described above. Ten raters were used for assessment of inter-rater reliability. To examine intra-rater reliability, evaluation of videotaped ADL was carried out twice, one month apart.

### Participants

#### Stroke patients

At five hospitals that provide stroke rehabilitation, the ADL of 43 stroke patients who agreed were video recorded. The inclusion criteria were as follows: (1) diagnosed with stroke according to the Classification of Cerebrovascular Diseases III of the National Institute of Neurological Disorders and Stroke [15]; (2) at least 20 years of age at the time of obtaining consent; (3) either sex; (4) patient or proxy fully understood the contents of this clinical study and freely agreed to participate; and (5) inpatients. The exclusion criteria were: (1) patients with comorbidities that could affect the evaluations in this study; (2) patients whose condition was unstable due to stroke; and (3) patients otherwise judged unfit for this study by the principal or other participating physicians.

#### Evaluators

Direct evaluation method: Direct observation for the validity study was performed by 10 physical or occupational therapists at the five hospitals where the video recording was conducted.

Video evaluation method: Video recordings of ADL were evaluated for the reliability study by 10 physical and occupational therapists (five each) from three hospitals that were different from the above five hospitals.

### PET-MBI

The PET-MBI is an evaluation sheet based on the MBI developed by Shah et al. that has been culturally adapted for use in Japan. For example, Japanese lifestyles (such as using chopsticks and taking a bath) are reflected in its evaluation processes. The PET-MBI has several features. It is a performance-based assessment (performance ADL), and it has increased usability, with decreased burden on the evaluators, since the minimum required explanations are provided on the evaluation sheet in consideration of its use in rehabilitation settings at facilities and at home in Japan. Furthermore, to enable anyone to perform evaluations in clinical settings, check boxes were provided for recording of information regarding the living environment of patients (such as availability of handrails).

As with the MBI, the highest score of the PET-MBI is 100, with higher scores indicating increased ADL. The scores are distributed among 10 items as follows: grooming and bathing (five points each); feeding, toilet use, stair climbing, dressing, bowel management, and bladder management (10 points each); and chair/bed transfer and mobility (15 points each). In a study targeting elderly individuals requiring care residing in care facilities, the PET-MBI showed high inter- and intra-rater reliabilities by two therapists. However, that study investigated the factorial validity of the PET-MBI using only nine items, excluding stair climbing [13]. This was because most older people in the care facilities did not climb stairs in their daily activities. The factorial validity of the PET-MBI was later verified using all 10 items in a different study targeting older people individuals requiring care living at home [14].

### Video recording of patients’ ADL and editing films

For the video evaluation method, video recordings of the ADL of each stroke patient were first made, and the resulting footage was then edited down to approximately 10 min per patient. In the selection of video data, the videos were viewed, and video footage with insufficient information was excluded based on the condition that stroke patients with wide ranges of level of independence be included. Of the 43 patients who participated in video production, 30 were chosen in consideration of the distribution of the levels of ADL independence (for the validity study and the reliability study). For video recording and editing, the general principles described below were followed.

1. Personal hygiene: Due to limitations in recording time, only tooth brushing was taped. (Tooth brushing was chosen because it is generally the most difficult grooming activity for stroke patients.)
2. Self-bathing: Patients were videotaped with their clothes on for their privacy.
3. Feeding: The first few minutes of a meal were recorded.
4. Using the toilet: Patients were videotaped with their clothes on for their privacy.
5. Stair climbing: “ADL capacity” was recorded; “ADL performance” data could not be obtained, since inpatients do not use stairs.
6. Getting dressed: Due to limitations in recording time, recording was only performed for either tops or bottoms (whichever was more difficult for the patient). (Patients’ ADL were not video recorded while they were putting on or removing orthoses.)
7. Bowel control: Only one scene of a nurse reporting the patient’s condition to a therapist was recorded.
8. Bladder control: Only one scene of a nurse reporting the patient’s condition to a therapist was recorded.
9. Chair/bed transfer: Due to limitations in recording time, patients’ transferring either from bed to a (wheel)chair or the reverse, whichever was more difficult for them, was recorded.
10. Ambulation: Videos were taken to demonstrate to the raters whether the patients were capable of walking 50 m.

### Data collection

The following basic information was collected from the stroke patients: sex, age, stroke history, classification of stroke [15], number of days from the onset of stroke to the start of video recording, disturbances of consciousness (on the Japan Coma Scale (JCS)) [16] during video recording, neurological disorders (such as dysarthria, sensation disorder, dysphagia, aphasia, agnosia, apraxia, and vision disorder), dominant hand, paralyzed side of the body, and cognitive function (on the Japanese version of the Mini Mental State Examination (MMSE-J)) [17, 18]. The basic information (sex, age, occupation, and number of years of clinical experience) of the therapists who conducted direct or video evaluation was also collected.

#### Validity study

For this study, physical or occupational therapists directly observed and scored patients’ ADL in a hospital environment.

#### Reliability study

Raters were trained in advance using videos of two patients with different levels of ADL independence that were not included in the actual study. For video evaluation study, 10 raters independently evaluated videos of 30 patients in their respective private rooms. The viewing order of these videos was randomized to avoid potential inter- and intra-rater biases.

On completion of the evaluations, the PET-MBI sheets were collected and sealed immediately. This eliminated the possibility of the raters exchanging opinions with other raters or correcting data.

### Statistical analysis

The basic information of the 30 stroke patients of whom video recordings were made of their ADL and then used for video evaluation, as well as the raters who conducted direct or video evaluation, was described. To verify the criterion-related validity of the PET-MBI against the BI, Pearson’s and Spearman’s correlation coefficients were calculated for the total scores obtained by the direct evaluation method. To determine the inter-rater reliability of the PET-MBI, Fleiss’ intraclass correlation coefficients (ICCs) were computed for the total scores from the first video evaluation as the primary outcome. As a secondary outcome, ICCs were determined for the total PET-MBI scores from the second video evaluation. Another secondary outcome was to calculate the kappa (κ) coefficients, weighted kappa (κ_w_) coefficients, and agreement rates of 10 PET-MBI category scores for each of the two video evaluation sessions.

For intra-rater reliability, the primary outcome was the ICCs of PET-MBI total scores, and the secondary outcome was the κ coefficients, κ_w_ coefficients, and agreement rates of 10 PET-MBI category scores, each calculated for the two sessions.

Statistical analysis was carried out using the Statistical Analysis System ver. 9.2 (SAS Institute Inc., Cary, NC, USA). Mean kappa coefficients were calculated using the number of raters. For inter-rater reliability, ICCs were computed by analysis of variance using all raters’ scores for each session. For intra-rater reliability, the mean ICC value for each rater was obtained.

ICCs and κ coefficients ≥0.61 were interpreted as “substantial,” and those ≥0.81 were interpreted as “almost perfect” [19].

### Ethical procedures

The study objectives and procedure were explained to participants or their legal representatives, and written consent was obtained from all participants. Approval was obtained from the Ethics Committees of Seijoh University (Approval number: 2013C0018), the affiliated institutions of all authors, and the hospitals where video recording was conducted. This study was registered with the University Hospital Medical Information Network Clinical Trials Registry (registration number: UMIN000013681).

## Results

Of the 30 stroke patients evaluated using both the direct and video evaluation methods, 23 (76.7%) were men. The mean age of all patients was 71.9 years, and 80.0% of them were ≥65 years old and over. The mean duration between the onset of stroke and video recording was 88.0 days. Table 1 shows the basic information for these patients. Both direct evaluation and video evaluation were conducted by physical and occupational therapists (five each) (Table 2).

**Table 1:** Characteristics of the 30 stroke patients

		Number of patients	%	Mean (standard deviation)
Sex	Male	23	76.7%	
	Female	7	23.3%	
Age (years)				71.9 (10.5)
Stroke history	First	22	73.3%	
	Recurrent	8	26.7%	
Classification of recent strokes	Cerebral hemorrhage	9	30.0%	
(May contain multiple entries per patient)	Subarachnoid hemorrhage	1	3.3%	
	Cerebral infarction	21	70.0%	
	Other	0	0.0%	
Number of days from the onset of stroke to the start of video recording	Mean (standard deviation)			88.0 (49.5)
Disturbances of consciousness during video recording (JCS)	0	0	0.0%	
	1 ~ 3	24	80.0%	
	10 ~ 30	6	20.0%	
	100 ~ 300	0	0.0%	
Movement disorder	Hemiplegia	27	90.0%	
(May contain multiple entries per patient)	Diplegia	1	3.3%	
	Ataxia	5	16.7%	
	Other	0	0.0%	
	None	1	3.3%	
Other neurological disorders	Dysarthria	13	43.3%	
(May contain multiple entries per patient)	Sensation disorder	18	60.0%	
	Dysphagia	11	36.7%	
	Aphasia	5	16.7%	
	Agnosia	9	30.0%	
	Apraxia	6	20.0%	
	Vision disorder	5	16.7%	
	Other	8	26.7%	
	None	2	6.7%	
Dominant hand	Left	0	0.0%	
	Right	30	100.0%	
Paralyzed side of the body	Left	15	50.0%	
	Right	14	46.7%	
	Both	1	3.3%	
	None	0	0.0%	
MMSE-J				19.5 (9.1)
BI total score (Direct evaluation)				52.0 (24.1)
PET-MBI total score (Direct evaluation)				52.7 (28.2)

*MMSE-J* Japanese version of the Mini Mental State Examination, *BI* Barthel Index, *PET-MBI* Performance evaluation tool based on the modified Barthel Index

**Table 2:** Characteristics of the raters

		Direct evaluation (10 raters)			Video evaluation (10 raters)		
		Number of raters	%	Mean (Standard deviation)	Number of raters	%	Mean (Standard deviation)
Sex	Male	7	70.0%		3	30.0%	
	Female	3	30.0%		7	70.0%	
Age (years)				32.1 (5.5)			41.3 (5.4)
Occupation	Physical therapist	5	50.0%		5	50.0%	
	Occupational therapist	5	50.0%		5	50.0%	
Clinical experience (years)				9.4 (5.8)			17.2 (6.4)

For the total scores obtained by the direct evaluation method, Pearson’s correlation coefficient of the BI versus the PET-MBI was 0.95, and the lower limit of the 95% confidence interval (CI) was 0.90. Identical values were obtained by Spearman’s rank correlation coefficient (Table 3).

**Table 3:** Correlation between the BI and the PET-MBI (for the total scores obtained by direct evaluation)

	Number of patients	Correlation coefficient	Lower limit of the 95% CI
Pearson	30	0.95	0.90
Spearman	30	0.95	0.90

For inter-rater reliability, the ICC using the total score of the first PET-MBI, which was the primary outcome, was 0.99, and that using the total score of the second PET-MBI, which was the secondary outcome, was also 0.99. When the scores of 10 PET-MBI items were independently analyzed, κ and κ_w_ coefficients were 0.61–0.89 and 0.77–0.94, respectively, for the first session, and 0.63–0.89 and 0.74–0.95, respectively, for the second session (Table 4).

**Table 4:** Inter-rater reliability of video evaluation

Primary outcome (PET-MBI total score)								
	Variance σ_s_^2^ of the subject effect	Variance σ_r_^2^ of the rater effect		Variance σ_E_^2^ of the error	Intraclass correlation coefficient (Fleiss’ ICC)		Lower limit of the 95% CI	
Session 1	742.81	1.47		8.42	0.99		0.98	
Session 2	732.19	3.65		7.67	0.99		0.97	
Secondary outcome (PET-MBI, mean κ coefficient, mean agreement rate)								
	Session 1				Session 2			
	κ_w_	κ	P_Ow_	P_O_	κ_w_	κ	P_Ow_	P_O_
Personal Hygiene	0.77	0.61	0.92	0.71	0.79	0.64	0.93	0.74
Self-bathing	0.80	0.70	0.94	0.81	0.74	0.64	0.92	0.75
Feeding	0.90	0.83	0.97	0.88	0.86	0.80	0.96	0.86
Using the Toilet	0.84	0.74	0.94	0.79	0.82	0.71	0.93	0.77
Stair Climbing	0.89	0.83	0.97	0.90	0.88	0.82	0.97	0.89
Getting dressed	0.87	0.69	0.94	0.75	0.84	0.63	0.93	0.70
Bowel Control	0.93	0.86	0.97	0.90	0.90	0.84	0.96	0.88
Bladder Control	0.94	0.89	0.97	0.92	0.93	0.89	0.97	0.92
Chair/Bed Transfer	0.85	0.77	0.95	0.82	0.86	0.78	0.95	0.84
Ambulation	0.91	0.74	0.97	0.79	0.95	0.79	0.98	0.82

*κ*_*w*_ Weighted κ coefficient (linear weights), *κ* κ coefficient, *P*_*Ow*_ Weighted agreement rate (linear weights), *P*_*O*_ Agreement rate

As the primary outcome of intra-rater reliability, an ICC of 0.99–1.00 was obtained for the PET-MBI total scores. Finally, the κ and κ_w_ coefficients of 10 PET-MBI item scores were independently calculated as the secondary outcome, and they were 0.78–0.92 and 0.85–0.96, respectively (Table 5).

**Table 5:** Intra-rater reliability of video evaluation

Primary outcome (PET-MBI total score)				
Rater identification number	Intraclass correlation coefficient (Fleiss’ ICC)			
Mean	0.99			
OT-1	0.99			
OT-2	1.00			
OT-3	1.00			
OT-4	1.00			
OT-5	0.99			
PT-1	0.99			
PT-2	1.00			
PT-3	0.99			
PT-4	1.00			
PT-5	0.99			
Secondary outcome (PET-MBI, mean κ coefficient, mean agreement rate)				
	κ_w_	κ	P_Ow_	P_O_
Personal Hygiene	0.90	0.81	0.97	0.86
Self-bathing	0.85	0.78	0.96	0.86
Feeding	0.91	0.85	0.97	0.90
Using the Toilet	0.91	0.85	0.97	0.88
Stair Climbing	0.94	0.91	0.98	0.95
Getting Dressed	0.94	0.86	0.97	0.89
Bowel Control	0.94	0.91	0.98	0.94
Bladder Control	0.95	0.92	0.98	0.94
Chair/Bed Transfer	0.92	0.87	0.97	0.90
Ambulation	0.96	0.88	0.98	0.90

*κ*_*w*_ Weighted κ coefficient (linear weights), *κ* κ coefficient, *P*_*Ow*_ Weighted agreement rate (linear weights), *P*_*O*_ Agreement rate

## Discussion

In this study, the correlation between the PET-MBI and the BI was analyzed first. When hospitalized stroke patients were directly assessed by these two measures, high correlation coefficients were obtained, strongly suggesting their concurrent validity. The reliability of the PET-MBI was then evaluated by applying it to stroke patients using a video evaluation technique. The results demonstrated high intra- and inter-rater reliabilities. This is consistent with a previous study conducted with elderly individuals [13]. Video evaluation techniques have been used in previous studies assessing ADL, which change over time, because they enable investigation of reliability through evaluation of ADL at a single point by multiple raters [20, 21]. In the present study as well, the use of this technique as a main evaluation method enabled assessment of PET-MBI in which 10 raters observed the same stroke patients.

The correlation coefficients of the total scores of the BI and the PET-MBI were high when the rater directly evaluated using these two measures. A correlation between the BI and the MBI has been reported elsewhere [22]. However, in the present study, there was also a correlation between the BI (ADL capacity) and the PET-MBI (ADL performance), which is an important finding.

In conducting this research, factors that influence the outcomes of raters’ evaluation were eliminated as much as possible. For example, the viewing orders of the ADL videos of the 30 patients were randomized for all raters, making it harder for them to share information. Similarly, the viewing orders were different for each rater between the first and second evaluation sessions, reducing a potential bias in the second session caused by the memory of the first. Thus, the fact that the PET-MBI still showed high inter- and intra-rater reliabilities strongly suggests the reliability of the present results. This high reliability of the PET-MBI may be attributable to the fact that its evaluation criteria are easy to understand, and the fact that the raters received training in advance using the manuals for this assessment tool. However, the manuals provided only the minimum necessary information, and their use was considered to be within acceptable standards of the reliability assessment studies of various evaluation tools. It has been recommended that the outcome measures of stroke patients have established psychometrics (reliability, validity, and sensitivity to change) [23]. Since the usability, reliability, and validity of the PET-MBI have now been established, we expect that this tool will be invaluable in clinical settings in evaluating stroke patients.

The inter-rater reliability for the item of grooming was “substantial”, but its κ coefficients were lower than those of the other items that mostly showed “almost perfect” reliability. The reason for this relatively low reliability may be related to the fact that therapists have fewer opportunities to observe patients’ grooming activities in everyday settings in rehabilitation at Japanese hospitals compared to other activities, as well as the fact that the evaluation criteria for this item were complex. Grooming includes several different activities. In this study, however, only tooth brushing was evaluated, and this activity was divided into the following three processes: preparation (put toothpaste on a toothbrush); execution (brushing); and completion (rinsing and tidying up). Despite these preparatory efforts, the reliability data for this particular item were less than ideal. As such, one must be careful when applying the PET-MBI to stroke patients in actual clinical settings.

There were some limitations in the present study. First, the only stroke patients targeted in this study were inpatients. In addition, we excluded any patient whose condition was unstable due to stroke, as well as those who were not suitable for video recording. These exclusions may have limited the generalizability of our findings. The use of PET-MBI should be considered for outpatients as well, and be scored by healthcare professionals other than therapists. Second, the raters had many years of clinical experience, and thus they may have been skilled at ADL scoring. However, they were unaccustomed to the evaluation styles used in this study. Thus, it is unlikely that their experience significantly affected the data. In addition, it was unclear whether the results were influenced by differences in sex, age, and duration of time practicing between those raters in the direct observation versus the video observation group. Although there were methodological differences between the validity study (direct observation) and the reliability study (video observation), we do not feel that these differences were likely to have influenced the results, since each of the total ICC scores was nearly perfect. Third, scores of the BI and the PET-MBI might have been mutually influential, due to the direct evaluation. However, BI measures the capacity of ADL (“can do”), while the PET-MBI measures performance of ADL (“do”). Moreover, we consider the mutual influence to be small, given the differences in observation scenes. Fourth, the raters were trained in advance using sample videos and manuals. This may have helped to produce consistently uniform evaluation results. However, the manuals provided only the minimum necessary information, and their use was considered to be within acceptable standards of the reliability assessment studies of various evaluation tools. Lastly, some might suggest that the reliability of the video evaluation technique increased because the videos were specifically edited to include sufficient information necessary for functional assessment. However, these videos showed only part of the patients’ ADL. Therefore, when compared with direct observation, the raters acquired much less information regarding patients’ functional dependency; this is precisely the reason that this scoring method was introduced into the protocol. Thus, it is unlikely that there would be significant bias in evaluation caused by video editing.

## Conclusions

The PET-MBI showed high concurrent validity when applied to stroke patients using the direct observation method and high intra- and inter-rater reliabilities in performing functional assessment by the video evaluation method. The PET-MBI is likely to become a convenient ADL evaluation tool that can be used by anyone.

## Appendix

Japanese version of performance evaluation tool of the MBI (Original web page for Department of Health Informatics, Kyoto University School of Public Health). URL: http://www.healthim.umin.jp/PET-BMI.html.

## Abbreviations

ADL: Activities of daily living
BI: Barthel Index
FIM: Functional Independence Measure
ICC: Intraclass correlation co-efficient
JCS: Japan Coma Scale
MBI: Modified Barthel Index
MMSE-J: Mini Mental State Examination, Japanese version
mRS: Modified Rankin Scale
PET-MBI: Performance evaluation tool of the MBI
SIAS: Stroke Impairment Assessment Set
